# Management of Infants in Hot Weather

**Published:** 1873-08

**Authors:** 


### SELECTIONS.

### MANAGEMENT OF INFANTS IN HOT WEATHER.

The following excellent rules for the care of infants during the hot months were prepared by a committee of six physicians appointed for the purpose by the Obstetrical Society of Philadelphia:

*Rule* 1.—Bathe the child once a day in tepid water. If it is feeble, sponge it all over twice a day with tepid water, or with tepid water and vinegar. The health of a child depends much upon its cleanliness.

*Rule* 2.—Avoid all tight bandaging. Make the clothing light and cool, and so loose that the child may have free play for its limbs. At night, undress it, sponge it, and put on a slip. In the morning, remove the slip and dress the child in clean clothes. If this cannot be afforded, thoroughly air the day-clothing by hanging it up during the night. Use clean diapers, and change them often. Never dry a soiled one in the nursery or in the sitting-room, and never use one for a second time without first washing it.

*Rule* 3.—The child should sleep by itself in a cot or cradle. It should be put to bed at regular hours, and be early taught to go to sleep without being nursed in the arms. Without the advice of a physician, never give it any *spirits, carminatives, soothing sirups, or sleeping drops. Thousands of children die every year from the use of these poisons.* If the child frets and does not sleep, it is either hungry or ill. If ill, it needs a physician. Never quiet it by candy or cake; they are the common causes of diarrhœa, and of other troubles.

*Rule* 4.—Give the child plenty of fresh air. In the cool of the morning and evening send it out to the shady sides of broad streets, to the public squares, or to the Park. Make frequent excursions on the rivers. Whenever it seems to suffer from the heat, let it drink freely of ice-water. Keep it out of the room in which washing or cooking is going on. It is excessive heat that destroys the lives of young infants.

*Rule* 5.—Keep your house sweet and clean, cool and well aired. In very hot weather, let the windows be open day and night. Do your cooking in the yard, in a shed, in the garret, or in an upper room. Whitewash the walls every spring, and see that the cellar is clear of all rubbish. Let no slops collect to poison the air. Correct all foul smells by pouring carbolic acid or quick-lime into the sinks and privies. The former article can be got from the nearest druggist, who will give the needful directions for its use.

Make every effort yourself, and urge your neighbors, to keep the gutters of your street or court clean.

*Rule* 6.—*Breast-milk is the only proper food for infants.* If the supply is ample, and the child thrives on it, no other kind of food should be given while the hot weather lasts. If the mother has not enough, she must not wean the child, but give it, besides the breast, goat's and cow's milk, as prepared under Rule 8. Nurse the child once in two or three hours during the day, and as seldom as possible during the night. Always remove the child from the breast as soon as it has fallen asleep. Avoid giving the breast when you are overfatigued or overheated.

*Rule* 7.—If, unfortunately, the child must be brought up by hand, it should be fed on a milk diet alone, and that warm milk out of a nursing bottle, as directed under Rule 8. Goat's milk is the best, and next to it cow's milk. If the child thrives on this diet, *no other kind of food whatever should be given while the hot weather lasts.* At all seasons of the year, but especially in summer, there is no safe substitute for milk to an infant that has not cut its front teeth. *Sago, arrow-root, potatoes, corn-flour, crackers, bread, every patented food, and every article of diet containing starch, can not and must not be depended on as food for very young infants.* Creeping or walking children must not be allowed to pick up unwholesome food.

*Rule* 8.—Each bottleful of milk should be sweetened by a small lump of loaf-sugar, or by half a teaspoonful of crushed sugar. If the milk is known to be pure, it may have one-fourth part of hot water need be added to it; but, if it is not known to be pure, no water need be added. When the heat of the weather is great, the milk may be given quite cold. Be sure that the milk is unskimmed; have it as fresh as possible, and brought very early in the morning. Before using the pans into which it is to be poured, always scald them with boiling suds. In very hot weather, boil the milk as soon as it comes, and at once put away the vessels holding it in the coolest place in the house—upon ice, if it can be afforded, or down a well. Milk carelessly allowed to stand in a warm room soon spoils, and becomes unfit for food.

*Rule* 9.—If the milk should disagree, a tablespoonful of lime-water may be added to each bottleful. Whenever pure milk can not be got, try the condensed milk, which often answers admirably. It is sold by all the leading druggists and grocers, and may be prepared by adding, without sugar, one teaspoonful, or more, according to the age of the child, to six tablespoonsful of boiling water. Should this disagree, a teaspoonful of arrow-root, of sago, or of corn starch, to the pint of milk may be cautiously tried. If milk in any shape can not be digested, try, for a few days, pure cream diluted with three-fourths or three-fifths of water—returning to the milk as soon as possible.

*Rule* 10.—The nursing bottle must be kept perfectly clean; otherwise the milk will turn sour, and the child will be made ill. After each meal it should be emptied, rinsed out, taken apart, and the tube, cork, nipple, and bottle be placed in clean water, or in water to which a little soda has been added. It is a good plan to have two nursing bottles, and to use them by turns.

*Rule* 11.—Do not wean the child just before or during the hot weather, nor, as a rule, until after its second summer. If suckling disagrees with the mother, she must not wean the child, but feed it in part, out of a nursing bottle, on such food as has been directed. However small the supply of breast milk, provided it agrees with the child, the mother should carefully keep it up against sickness; it alone will often save the life of a child when every thing else fails. When the child is over six months old, the mother may save her strength by giving it one or two meals a day of stale bread and milk, which should be pressed through a seive and put into a nursing bottle. When from eight months to a year old, it may have also one meal a day of the yolk of a fresh and rare-boiled egg, or one of beef or mutton broth into which stale bread has been crumbled. When older than this, it can have a little meat finely minced; but even then milk should be its principal food, and not such food as grown-up people eat.

For the convenience of mothers, the following receipts for special forms of diet are given:

*Boiled Flour, or Flour Ball.*—Take one quart of good flour, tie it up in a pudding-bag so tightly as to get a firm, solid mass, put it into a pot of boiling water early in the morning, and let it boil until bedtime. Then take it out and let it dry. In the morning, peel off from the surface and throw away the thin rind of dough, and, with a nutmeg-grater, grate down the hard, dry mass into a powder, Of this, from one to three teaspoonsful may be used, by first rubbing it into a paste with a little milk, then adding it to about a pint of milk, and, finally, by bringing the whole to just the boiling point. It must be given through a nursing bottle.

An excellent food for children who are costive in their bowels may be made by using bran-meal or unbolted flour instead of the white flour, preparing it as above directed.

*Rice Water.*—Wash four tablespoonfuls of rice, put it into two quarts of water, which boil down to one quart, and then add sugar and a little nutmeg. This makes a pleasant drink.

A half pint or a pint of milk added to this, just before taking it from the fire, and allowed to come to a boil, gives a nourishing food suitable for cases of diarrhœa.

Sago, tapioca, barley, or cracked corn can be prepared in the same manner.

*Beef Tea.*—Take one pound of juicy, lean beef—say a piece off the shoulder or the round—and mince it up with a sharp knife on a board or a mincing-block. Then put it with its juice into an *earthen* vessel containing a pint of tepid water, and let it stand for two hours. Strain off the liquid through a clean cloth, squeezing well the meat, and add a little salt. Place the whole of the juice thus obtained over the fire, but remove it as soon as it has become browned. Never let it boil; otherwise most of the nutritious matter of the beef will be thrown down as a sediment. A little pepper or allspice may be added if preferred.

Mutton-tea may be prepared in the same way. It makes an agreeable change when the patient has become tired of beef-tea.

*Raw Beef for Children.*—Take half a pound of juicy beef, free from any fat; mince it up very finely; then rub it into a smooth pulp, either in a mortar or in an ordinary potato-masher. Spread a little out upon a plate and sprinkle over it some salt, or some sugar, if the child prefers it. Give it with a teaspoon or upon a buttered slice of stale bread. It makes an excellent food for children with dysentery.

